# Supplementary material for: Reduced Amino Acid Substitution Matrices Find Traces of Ancient Coding Alphabets in Modern Day Proteins
Source: Mol Biol Evol. 2025 Aug 11;42(9):msaf197. doi: 10.1093/molbev/msaf197 (PMC12402984; doi:10.1093/molbev/msaf197)
Supplement: msaf197_Supplementary_Data [file msaf197_supplementary_data.pdf]

Supporting information: Reduced amino acid substitution models find traces of ancient coding alphabets in modern day proteins

Jordan Douglas[1,2,*] ORCID: 0000-0003-0371-9961
Remco Bouckaert[2,3] ORCID: 0000-0001-6765-3813
Charles W. Carter, Jr[4] ORCID: 0000-0002-2653-4452
Peter R. Wills[1,2,5] ORCID: 0000-0002-2670-7624

[1]Department of Physics, The University of Auckland, New Zealand
[2]Centre for Computational Evolution, The University of Auckland, New Zealand
[3]School of Computer Science, The University of Auckland, New Zealand
[4]Department of Biochemistry and Biophysics, University of North Carolina at Chapel Hill, USA
[5]Integrative Transcriptomics, Interfaculty Institute
for Bioinformatics and Medical Informatics (IBMI), University of Tübingen, Germany
*jordan.douglas@auckland.ac.nz
Keywords: genetic code; aminoacyl-tRNA synthetases; phylogenetics; molecular evolution

June 12, 2025

Contents

1 Simulation studies **2**
1.1 Coverage simulation studies 2
1.2 Bayesian model averaging 5
1.3 Estimating tree height bias on simulated data 7

2 An empirical substitution model of the aaRS **9**

3 Biological datasets **11**
3.1 Prior distributions 11
3.2 Site compositions 12

4 Joint aaRS phylogenetic analysis **14**
4.1 Prior distributions for calibrated root analysis (r1) 14
4.2 Prior distributions for uncalibrated root analysis (r2) 15

1 Simulation studies

1.1 Coverage simulation studies

We performed coverage simulation studies[1] to validate our phylogenetic model. This involved the following steps, across 100 replicates. First, we simulated a set of binary rooted time-trees $\mathcal{T}$ as a Yule process, with birth rate λ sampled from its prior distribution. These trees were simulated such that the average tree height was around 1.5 substitutions per site to achieve mutational saturation. Second, we selected the substitution model indicator $\mathbb{I}_s \in \{0, 1, 2, 3\}$. This step was not stochastic - rather we ensured that $\mathbb{I}_s = 0$ on 50/100 replicates, $\mathbb{I}_s = 1$ on 25/100, and $\mathbb{I}_s = 2$ on 13/100, and $\mathbb{I}_s = 3$ on 12/100. Third, we uniformly at random sampled a cherry $\alpha\beta \in \{$AG, DN, DK, EQ, FH, IV, PT, SG, WY, NQ, CF, LS, DE, FY$\}$. Fourth, we simulated amino acid sequences down the tree $\mathcal{T}$ using the `resub`$(\alpha\beta)$ model, including model indicator $\mathbb{I}_s$. We used the aaRS substitution model (Table S1 and Fig. S5) and the optimised relaxed clock model[2] during this step. Fifth, we performed MCMC on each simulated dataset to estimate the tree and its parameters. Lastly, we compared the known values of parameters with their posterior estimates.

We validated this approach using coverage simulation studies with $N = 20$ (Fig. S1) and $N = 40$ (Fig. S2) taxa. These experiments confirmed the true value of a parameter lies in its 95% credible interval approximately 95% of the time; thereby providing confidence in the correctness of our method, and its ability to recover parameter estimates from data simulated under a known model.

During these experiments, the following priors were used:

- Birth rate
  - $\lambda \sim \text{LogNormal}(\text{mean} = 2, \sigma = 0.2)$ when $N = 20$
  - $\lambda \sim \text{LogNormal}(\text{mean} = 3, \sigma = 0.2)$ when $N = 40$
- Gradual relaxed clock standard deviation $\sim \text{Gamma}(\alpha = 5, \beta = 0.05)$
- Gamma rate heterogeneity shape $\sim \text{Exponential}(\mu = 1)$
- Transition proportion $\nu \sim \text{Beta}(\alpha = 2, \beta = 2)$
- Relative transition age $\frac{t_e}{t_h} \sim \text{Beta}(\alpha = 2, \beta = 2)$ where t_h is the root height
- Amino acid equilibrium frequencies $\pi \sim \text{Dirichlet}(\alpha_1 = 4, \alpha_2 = 4, \dots, \alpha_{20} = 4)$ - and fixed to the aaRS empirical substitution model during simulation (Table S1).
- Amino acid exchangeability relative rates $\mathbf{r} \sim \text{LogNormal}(\text{mean} = 1, \sigma = 1)$ - and fixed to the aaRS empirical substitution model during simulation (Fig. S5).
- Model indicator $\mathbb{I}_s = \begin{cases} 0 & \text{w.p. } \frac{1}{2} \\ 1 & \text{w.p. } \frac{1}{4} \\ 2 & \text{w.p. } \frac{1}{8} \\ 3 & \text{w.p. } \frac{1}{8} \end{cases}$

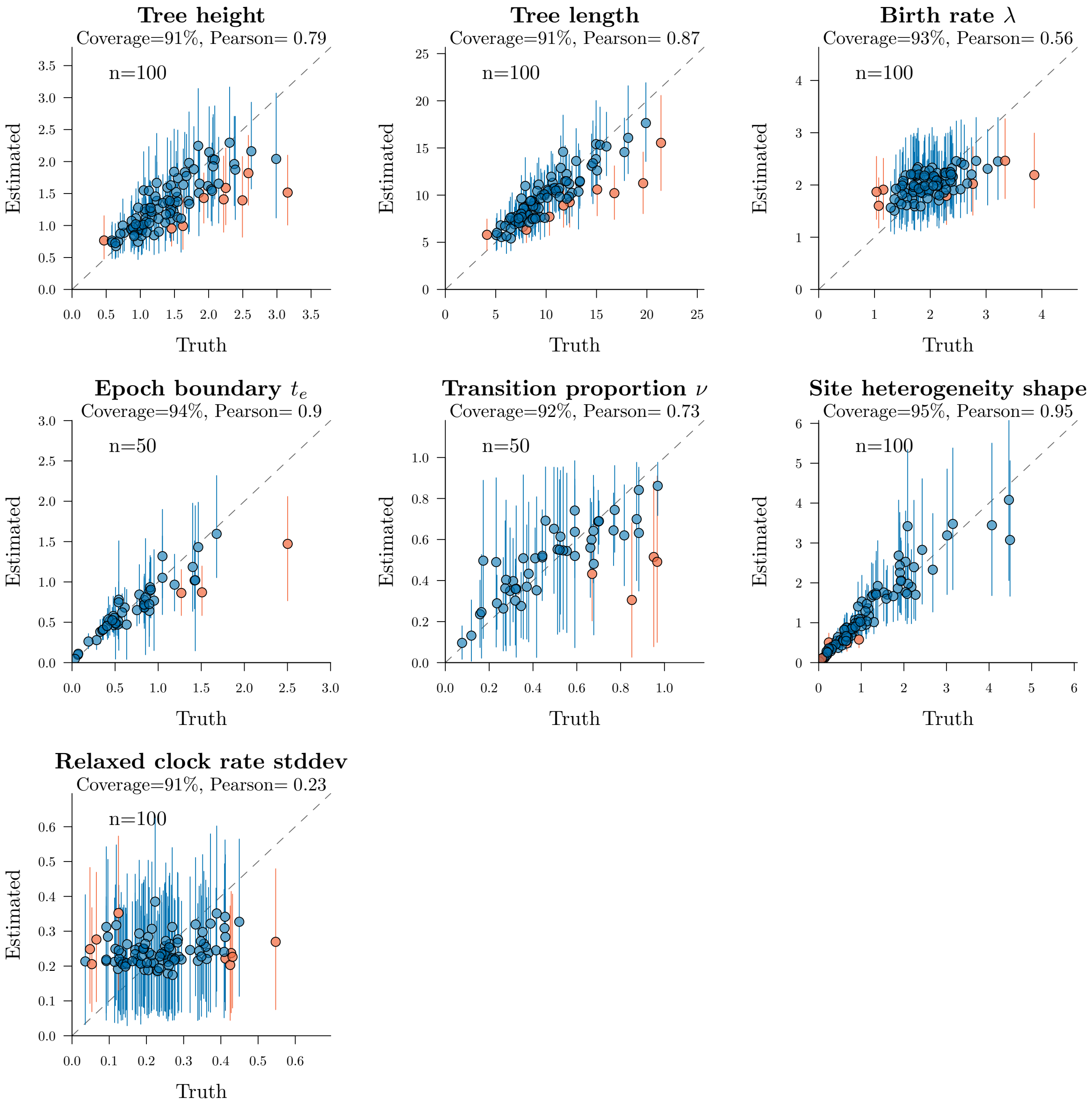

Fig. S1: Well-calibrated simulation study on trees with $N = 20$ taxa and $L = 50$ amino acid sites under the `resub` model. $n = 100$ replicates were performed in total, with t_e and ν plots being conditional on the true model indicator $\mathbb{I}_s > 0$. Points are coloured blue if the true value is within the 95% credible interval, and red otherwise. The coverage is the proportion of replicates whose true value is within the 95% credible interval (close to 95% indicating good coverage). The Pearson correlation between true values and mean estimates is also reported.

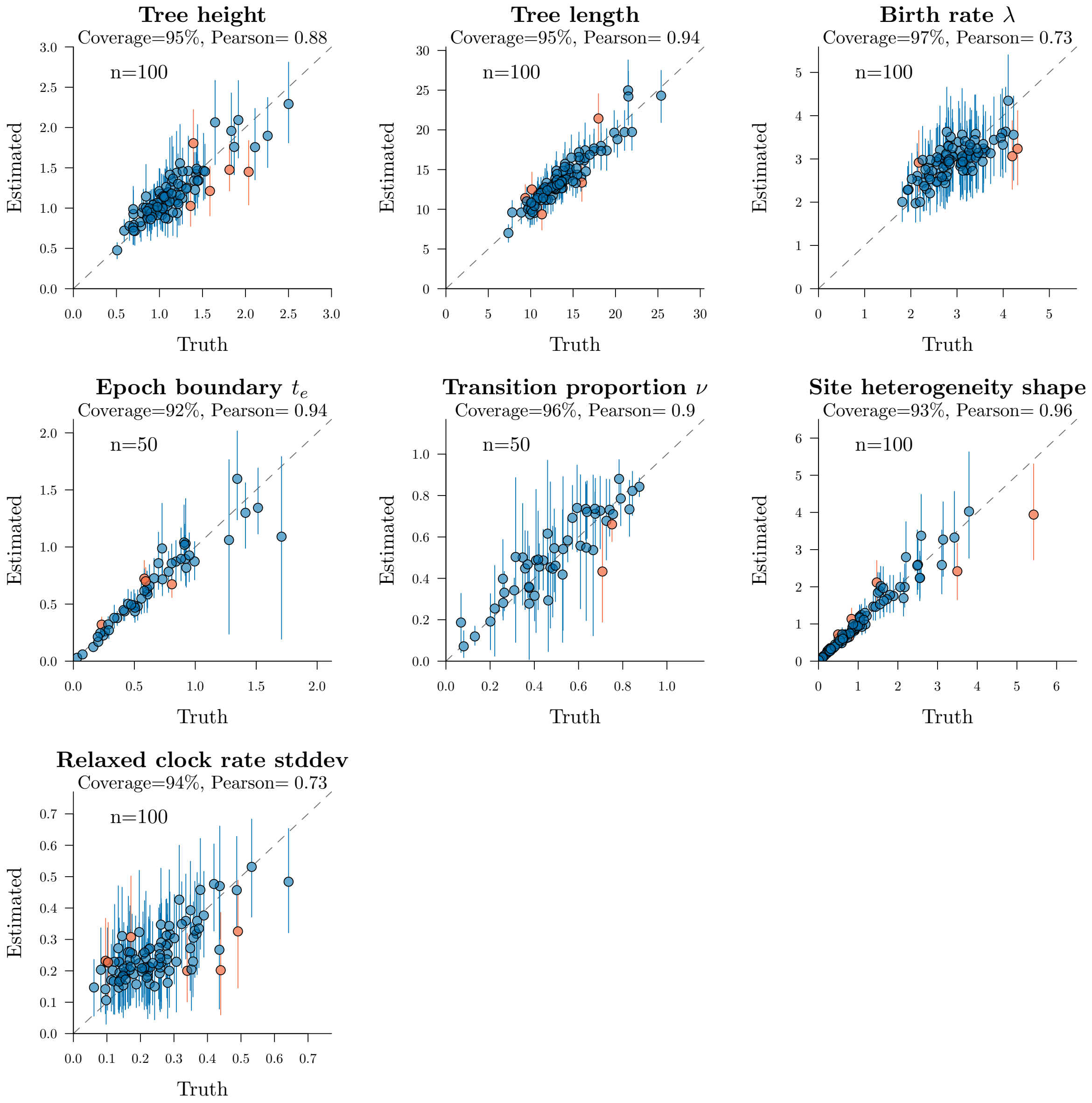

Fig. S2: Well-calibrated simulation study on trees with $N = 40$ taxa and $L = 200$ amino acid sites. Refer to Fig. S1 for figure notation.

1.2 Bayesian model averaging

By performing additional simulation studies, we characterised how accurately our new method can identify the true substitution model $\mathbb{I}_s$ on data simulated under a known sibstituiton model. The simulations were conducted under the same conditions described in the section above, for varying alignment lengths $L = (50, 100, 200)$ and taxon counts $N = (20, 40)$. As shown in Fig 3 of the main article and Fig. S3 here, this method can usually recover the true `resub` model (and whether it is appropriate at all), with high confidence even on small datasets. These experiments confirm this approach is statistically consistent, with accuracy improving with both longer alignments and larger trees. The method was seldom wrong on simulated data.

We compared varying hypotheses using Bayes factors. Following the guidelines of Kass and Raftery,[3] a Bayes factor B_h of 10 indicates "strong" support in favour of hypothesis h. This threshold corresponds to the following posterior probabilities:

$$B_h = \frac{p(\mathbb{I}_s = h|D)}{p(\mathbb{I}_s = h)} \div \frac{p(\mathbb{I}_s \neq h|D)}{p(\mathbb{I}_s \neq h)} \tag{1}$$

$$\begin{aligned} \Rightarrow p(\mathbb{I}_s = 0|D) &> 0.91 \text{ when } B_0 > 10. \\ p(\mathbb{I}_s = 1|D) &> 0.77 \text{ when } B_1 > 10. \\ p(\mathbb{I}_s = 2|D) &> 0.59 \text{ when } B_2 > 10. \\ p(\mathbb{I}_s = 3|D) &> 0.59 \text{ when } B_3 > 10. \end{aligned} \tag{2}$$

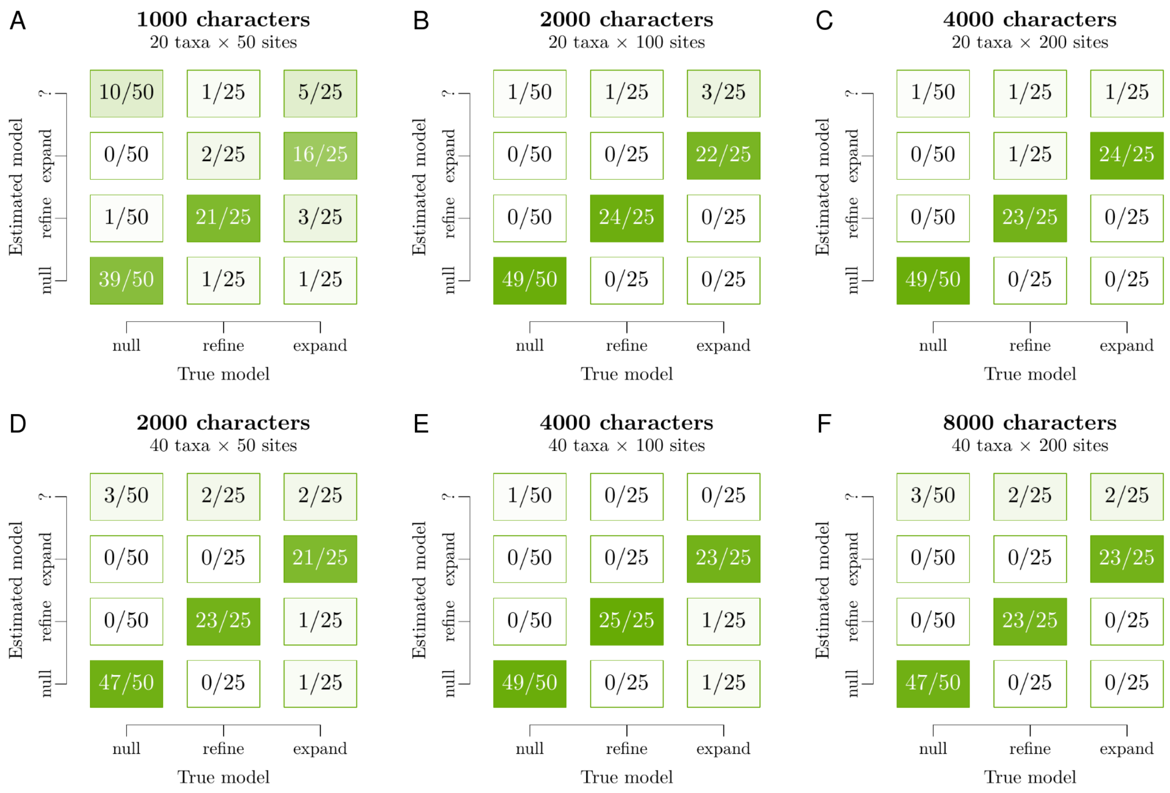

Fig. S3: Bayesian model averaging on simulated data of varying sizes. 100 datasets were simulated and 100 MCMC chains run for each panel in the figure, and the inferred model was classified into no resub (i.e., null), refinement, expansion, or unclassified using a Bayes factor threshold of 10. These results confirm that the method is rarely wrong, and can often recover the true resub model (refinement or expansion) with high confidence even with limited data availability. Note that this figure is an alternative view of the same data presented in Fig. 3 of the main article.

1.3 Estimating tree height bias on simulated data

In Fig. 7 of main article, we assessed the impact of applying either model ($\mathbb{I}_s$ fixed at 0, or fixed at its true value > 0) to data generated under the latter. A further breakdown of this experiment is presented in Fig. S4. We showed that the former produced biased estimates of tree height and the age of the old epoch. These trees were calibrated time trees, meaning that the node ages represented time units informed by the presence of sampled ancestral nodes on the tree. Sampled ancestors were simulated under a birth-death-sampling model[4] with the sampling proportion fixed to 0.3. The simulated alignments consisted of $L = 200$ amino acid sites, $N = 40$ extant taxa, and a variable number of non-extant taxa (mean: 24, 95% HPD: (2, 67), range: (2, 169)). Data were simulated and inferred under a relaxed clock model.[2] The following priors were used in this experiment:

- Clock rate
  - $\mu_C = 1$ during simulation
  - $\mu_C \sim \text{LogNormal}(\text{mean} = 1, \sigma = 1)$ during inference
- Birth rate
  - $\lambda \sim \text{LogNormal}(\text{mean} = 2, \sigma = 0.2)$ during simulation
  - $\lambda \sim \text{LogNormal}(\text{mean} = 10, \sigma = 2)$ during inference (i.e., an uninformed prior)
- Reproduction number $\frac{\lambda}{\mu} - 1 \sim \text{Exponential}(\text{mean} = 1)$
- Gradual relaxed clock standard deviation $\sim \text{Gamma}(\alpha = 5, \beta = 0.05)$
- Gamma rate heterogeneity shape $\sim \text{Exponential}(\mu = 1)$
- Transition proportion $\nu \sim \text{Beta}(\alpha = 5, \beta = 5)$
- Relative transition age $\frac{t_e}{t_h} \sim \text{Beta}(\alpha = 10, \beta = 2)$
- Model indicator
  - $\mathbb{I}_s = \begin{cases} 0 & \text{w.p. } 0 \\ 1 & \text{w.p. } \frac{1}{2} \\ 2 & \text{w.p. } \frac{1}{4} \\ 3 & \text{w.p. } \frac{1}{4} \end{cases}$ during simulation
  - $\mathbb{I}_s$ fixed at either 0 or the true value during inference
- Amino acid equilibrium frequencies $\sim \text{Dirichlet}(\alpha_A = 4, \alpha_C = 4, \ldots, \alpha_Y = 4)$
- Amino acid exchangeability relative rates fixed to the aaRS empirical substitution model

Under this prior distribution, the true tree height t_h averaged 3.2 substitutions per site (95% credible interval: (1.2, 6.5)). However, when doing inference, we gave λ an uninformed prior, which resulted in the tree height averaging 34 (0.009, 126) substitutions per site. As a result, the tree height during inference was informed by the data, and not by the prior.

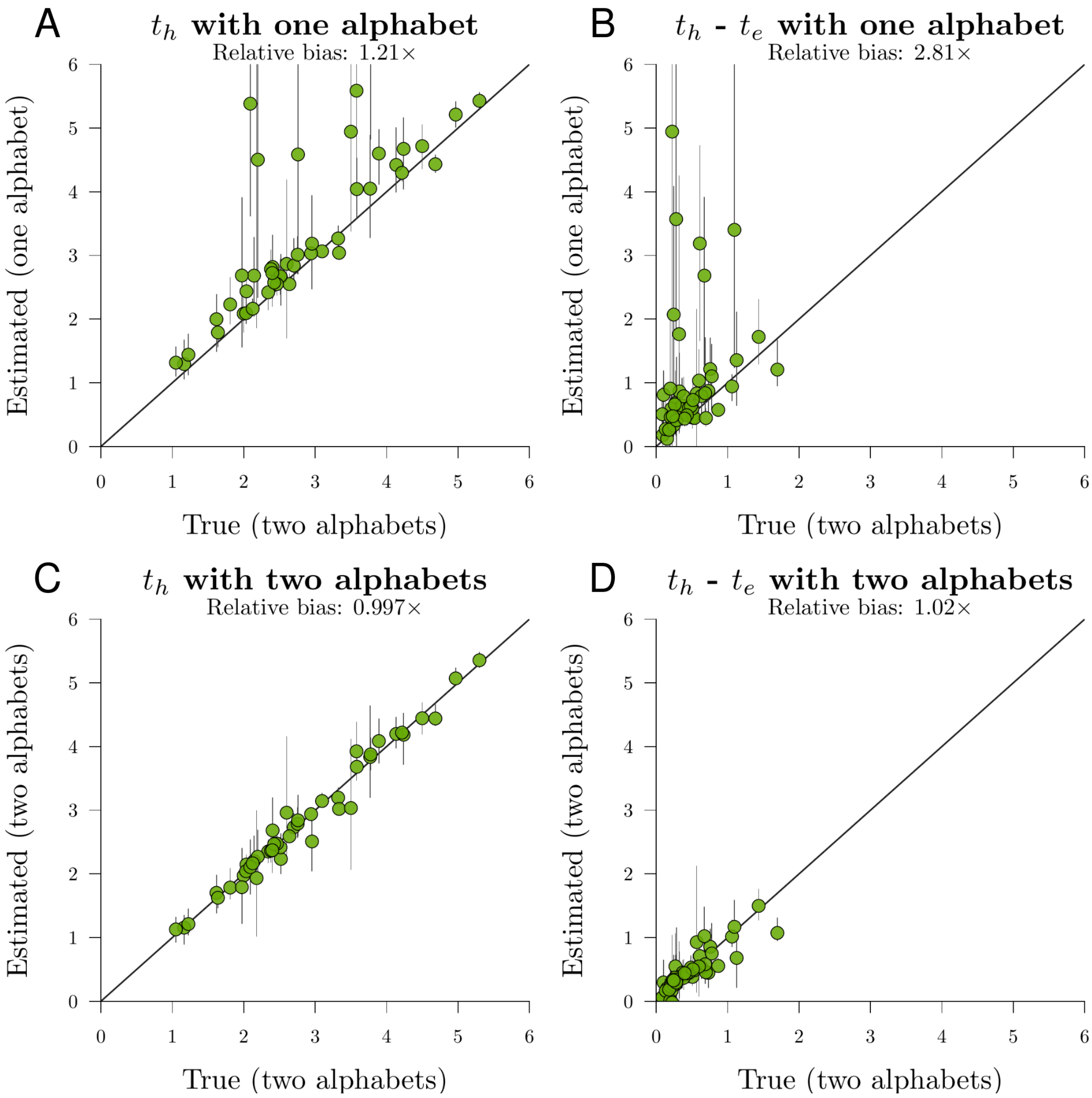

Fig. S4: Each point represents one of the fifty datasets simulated under the two-alphabet model. Inference was performed using either one alphabet (A and B) or two alphabets (C and D). These experiments confirm that application of the true substitution model gives unbiased estimates of the tree height, while the assumption of having just one alphabet gives an overestimate. Note that A and B are displaying the same data presented in Fig. 7 panels C and D of the main article.

2 An empirical substitution model of the aaRS

We estimated the exchangeability matrix $\mathbf{r}$ from a joint Bayesian analysis of the Class I and II aaRS catalytic domains. This matrix was used for simulating data in our well-calibrated simulation studies. This matrix is shown in Fig. S5. The estimated frequencies are shown in Table S1.

Amino acid	Estimated frequency
A	0.07611
C	0.01206
D	0.04293
E	0.07649
F	0.05826
G	0.06448
H	0.02521
I	0.0684
K	0.05763
L	0.09824
M	0.02922
N	0.03194
P	0.03257
Q	0.03192
R	0.06107
S	0.0547
T	0.04757
V	0.06342
W	0.02267
Y	0.04511

Table S1: Estimated amino acid frequencies in the Class I and II aaRS catalytic domains.

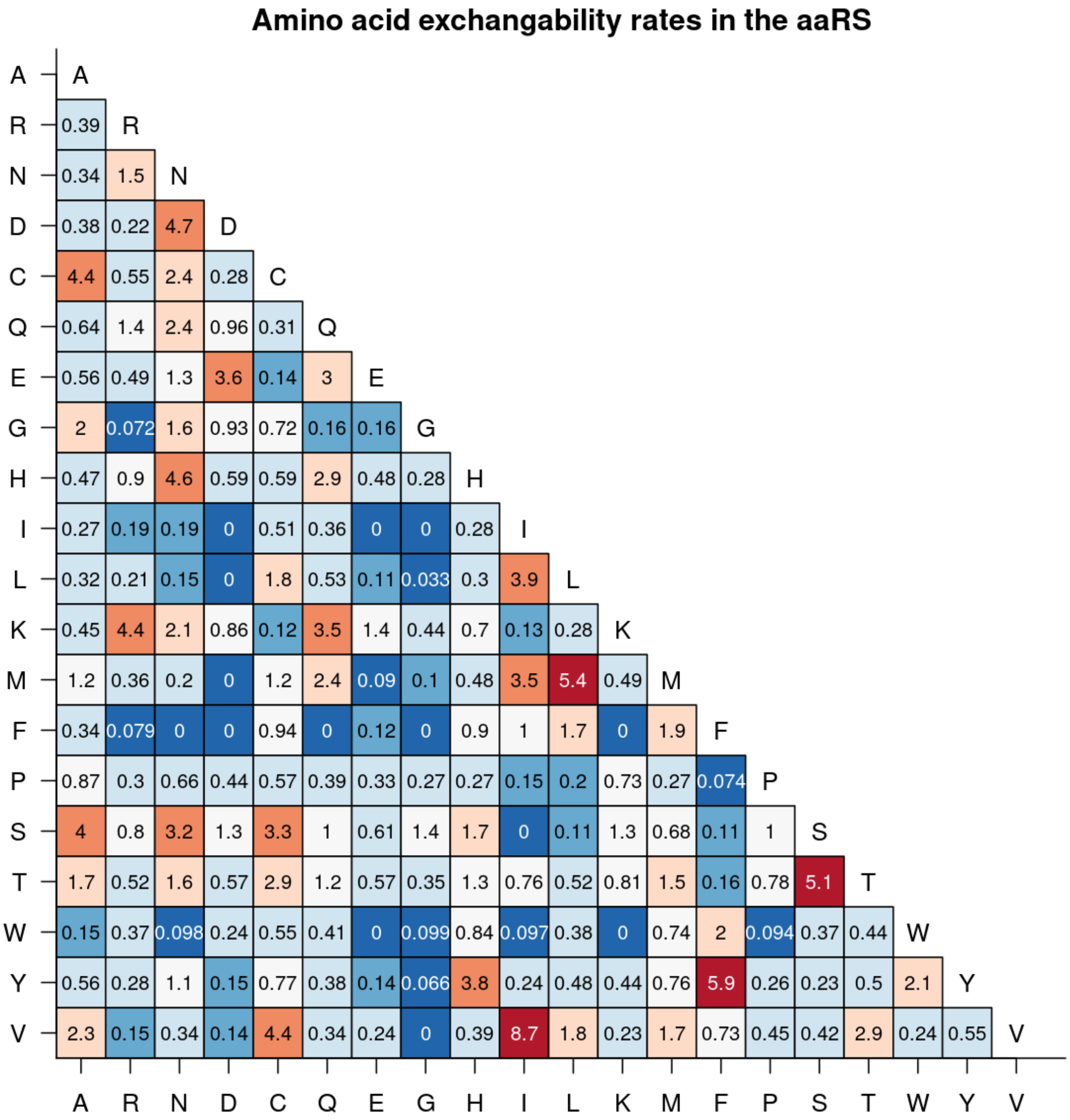

Fig. S5: Amino acid exchangeability matrix estimated from the Class I and II aaRS catalytic domains. The numbers shown are relative transition rates, with reds indicating faster rates, and blue indicating slow rates. The 0 rates correspond to parameters that were deemed as unnecessary by stochastic variable selection. The values shown here are median posterior estimates.

3 Biological datasets

3.1 Prior distributions

The following prior distributions were used to generate the resub screening results presented in Fig. 4 of the main article:

- Birth rate $\lambda \sim \text{LogNormal}(\text{mean} = 1, \sigma = 2)$
- Reproduction number $\frac{\lambda}{\mu} - 1 \sim \text{Exponential}(\text{mean} = 5)$
- Gradual relaxed clock standard deviation $\sim \text{Gamma}(\alpha = 5, \beta = 0.05)$
- Gamma rate heterogeneity shape $\sim \text{Exponential}(\mu = 1)$
- Amino acid equilibrium frequencies $\sim \text{Dirichlet}(\alpha_A = 4, \alpha_C = 4, \dots, \alpha_Y = 4)$
- Amino acid exchangeability rates $\mathbf{r} \sim \text{LogNormal}(\text{mean} = 1, \sigma = 1)$
- Transition proportion $\nu \sim \text{Beta}(\alpha = 4, \beta = 4)$
- Relative transition age $\frac{t_e}{t_h} \sim \text{Beta}(\alpha = 6, \beta = 2)$
- Model indicator $\mathbb{I}_s = \begin{cases} 0 & \text{w.p. } \frac{1}{2} \\ 1 & \text{w.p. } \frac{1}{4} \\ 2 & \text{w.p. } \frac{1}{8} \\ 3 & \text{w.p. } \frac{1}{8} \end{cases}$

3.2 Site compositions

To better understand these biological datasets, we counted the number of alignment sites that contain both members of each cherry (Table S2) and the empirical frequency of each amino acid (Table S3). These observations confirm that each amino acid and each cherry were well-represented across the datasets.

Dataset	AG	SG	IV	PT	DK	DN	EQ	FH	WY	LS	NQ	CF	DE	FY
Transferrin	65	75	74	36	70	74	83	8	7	55	49	7	89	28
Papain-like protease	71	76	75	55	75	79	72	25	12	76	61	9	84	43
Trypsin	135	151	114	110	108	101	109	55	34	176	90	35	117	72
Carbonic anhydrase	125	129	109	78	94	96	108	38	14	142	79	14	115	40
Cytochrome P450	151	124	117	82	103	88	121	45	21	112	71	18	143	55
I3G phosphate synthase	49	39	77	17	56	42	58	8	4	42	31	7	57	20
DNA repair	113	130	172	90	185	150	170	47	24	215	115	29	217	74
GST	68	67	82	39	63	56	66	43	16	69	52	19	71	55
Elongation factors	125	90	229	65	155	99	123	22	12	120	63	12	184	43
TPI	99	97	112	49	81	88	87	41	10	94	89	24	85	43
Class I urzyme bact.	71	81	90	49	53	57	54	54	31	91	60	42	61	76
Class I urzyme	107	111	115	78	81	85	90	81	54	119	96	69	91	99
Class I cat	127	130	133	90	104	101	112	100	81	134	114	88	111	119
Class II urzyme bact.	95	89	97	63	73	81	95	63	42	106	90	43	89	74
Class II urzyme	123	126	128	98	103	118	125	103	75	134	123	76	123	112
Class II cat	133	132	132	94	96	113	108	107	92	136	109	88	112	122

Table S2: The number of alignment sites that contain both members of each cherry.

Dataset	A	C	D	E	F	G	H	I	K	L	M	N	P	Q	R	S	T	V	W	Y
Transferrin	0.0904	0.047	0.0618	0.0586	0.0367	0.0789	0.0206	0.0368	0.0754	0.0823	0.014	0.0382	0.0435	0.0358	0.0375	0.0774	0.0525	0.0646	0.0127	0.0351
Papain-like protease	0.0759	0.0342	0.051	0.0587	0.0332	0.0978	0.0218	0.0538	0.059	0.0566	0.016	0.0545	0.0408	0.0371	0.0386	0.0775	0.0527	0.0677	0.0238	0.0494
Trypsin	0.0674	0.0447	0.0478	0.0446	0.0252	0.0916	0.0308	0.0464	0.042	0.0882	0.0159	0.0412	0.0597	0.042	0.0474	0.0785	0.0535	0.0753	0.0241	0.0336
Carbonic anhydrase	0.06	0.0121	0.0515	0.0653	0.0377	0.0664	0.0359	0.0439	0.0469	0.0959	0.018	0.0469	0.0643	0.0455	0.0424	0.0878	0.0603	0.0664	0.0172	0.0355
Elongation factors	0.0721	0.011	0.0539	0.0839	0.0299	0.0807	0.0212	0.0825	0.0866	0.0776	0.0206	0.0341	0.0518	0.0282	0.0494	0.0439	0.0547	0.0882	0.00765	0.0221
I3G phosphate synthase	0.1	0.0079	0.0578	0.0933	0.032	0.0569	0.0106	0.0787	0.0663	0.111	0.0164	0.0324	0.0386	0.0295	0.0646	0.0634	0.0355	0.0769	0.00332	0.0247
DNA repair	0.0686	0.00872	0.0663	0.0864	0.0384	0.0567	0.0146	0.063	0.084	0.102	0.0201	0.0428	0.0451	0.0422	0.0542	0.0683	0.0458	0.0553	0.00808	0.0296
Cytochrome P450	0.0958	0.0106	0.0669	0.0726	0.0447	0.0622	0.0284	0.0483	0.0338	0.115	0.0246	0.0257	0.0625	0.0343	0.0789	0.0483	0.0524	0.0658	0.00841	0.0203
Glutathione S-transferase	0.0809	0.00726	0.0546	0.0799	0.05	0.0553	0.0218	0.0536	0.0816	0.115	0.0284	0.0354	0.0485	0.0367	0.0467	0.0469	0.0376	0.0657	0.0118	0.042
Triosephosphate isomerase	0.108	0.0154	0.0433	0.073	0.0331	0.0811	0.0229	0.0815	0.0591	0.0849	0.0186	0.0474	0.0316	0.0422	0.0378	0.0617	0.0471	0.076	0.0114	0.0242
Class I urzyme bacterial	0.0646	0.0105	0.0592	0.0371	0.053	0.087	0.0436	0.0643	0.0527	0.0914	0.0286	0.0393	0.05	0.028	0.0499	0.0558	0.0531	0.0666	0.0175	0.0476
Class I urzyme	0.0654	0.0113	0.057	0.0386	0.0541	0.0823	0.0442	0.0654	0.0549	0.091	0.0286	0.0377	0.0498	0.0275	0.05	0.0584	0.0525	0.0666	0.0181	0.0468
Class I catalytic domain	0.0687	0.0131	0.0504	0.0524	0.0581	0.06	0.0406	0.0673	0.0616	0.09	0.0288	0.0346	0.0421	0.0324	0.0557	0.0579	0.0441	0.0638	0.0231	0.0551
Class II urzyme bacterial	0.0693	0.01	0.0467	0.086	0.0633	0.0592	0.03	0.0558	0.0468	0.0979	0.0308	0.0344	0.0441	0.0407	0.0698	0.0471	0.0547	0.0585	0.0127	0.0422
Class II urzyme	0.0647	0.0119	0.0467	0.0859	0.0655	0.0553	0.0302	0.0584	0.0495	0.0997	0.0312	0.0349	0.0418	0.0388	0.0672	0.0511	0.0534	0.0591	0.0128	0.0419
Class II catalytic domain	0.0629	0.0155	0.0407	0.0828	0.0636	0.0698	0.0256	0.0698	0.0422	0.103	0.0338	0.0312	0.0363	0.0348	0.0619	0.0501	0.0516	0.0668	0.015	0.0426

Table S3: Empirical amino acid frequencies in each dataset (rounded to 3 sf).

4 Joint aaRS phylogenetic analysis

4.1 Prior distributions for calibrated root analysis (r1)

The following prior distributions were used in our Class I and II aaRS catalytic domain joint analyses, presented in Fig. 5 and 6 of the main article. This model featured a birth-death tree prior and a gamma spike clock model.[5] Under this model, the overall evolutionary rate of a Class tree is equal to the clock rate times the relative mutation rate of that tree.

- Birth rates; one per Class $\lambda \sim \text{LogNormal}(\text{mean} = 1, \sigma = 1)$
- Reproduction numbers; one per Class $\frac{\lambda}{\mu} - 1 \sim \text{Exponential}(\text{mean} = 5)$
- Gradual relaxed clock standard deviations; one per Class $\sim \text{Gamma}(\alpha = 5, \beta = 0.05)$
- Spike means; one per Class $S_\mu \sim \text{LogNormal}(\text{mean} = 0.01, \sigma = 1.2)$
- Spike shapes; one per Class $S_\alpha \sim \text{LogNormal}(\text{mean} = 2, \sigma = 0.5)$
- Clock rate; amino acid substitutions per site per billion years $\sim \text{LogNormal}(\text{mean} = 0.1, \sigma = 1)$
- Relative mutation rates; one per Class $\sim \text{LogNormal}(\text{mean} = 1, \sigma = 1)$
- Gamma rate heterogeneity shapes; one per Class $\sim \text{LogNormal}(\text{mean} = 2, \sigma = 0.5)$
- Amino acid equilibrium frequencies $\sim \text{Dirichlet}(\alpha_A = 4, \alpha_C = 4, \ldots, \alpha_Y = 4)$
- Amino acid exchangeability rates $\mathbf{r} \sim \text{LogNormal}(\text{mean} = 1, \sigma = 1)$
- Transition proportion $\nu \sim \text{Beta}(\alpha = 5, \beta = 5)$
- Relative transition age $t_e - t_a \sim \text{Laplace}(\lambda = 100)$, where t_a is the estimated height of the cherry aaRS ancestor.
- Model indicator $\mathbb{I}_s = \begin{cases} 0 & \text{w.p. } \frac{1}{2} \\ 1 & \text{w.p. } \frac{1}{4} \\ 2 & \text{w.p. } \frac{1}{8} \\ 3 & \text{w.p. } \frac{1}{8} \end{cases}$

The transition boundary t_e was *a priori* constrained to sensible estimates informed by the aaRS phylogeny. In the general case, t_e was strongly constrained to occur near the same height t_a as the respective aaRS node. In the case of real cherries, this is straightforward. For example, in the WY cherry, t_a is the time of the ancestor of TrpRS and TyrRS. In the case of the fake cherries, t_a was linked to one of the other cherry ancestors, chosen arbitrarily. For LS, t_a was the time of the ThrRS/ProRS ancestor; GluRS/GlnRS for CF; SerRS/GlyRSlnRS for NQ; IleRS/ValRS for DE, and TyrRS/TrpRS for FY.

4.2 Prior distributions for uncalibrated root analysis (r2)

In the aaRS analysis of Fig. 7 of the main article, we removed the root age divergence time prior, but retained the other calibration priors (LUCA, LBCA, LACA, LMCA, and LECA). We applied a relaxed clock[2] and a birth-death-skyline tree prior.[6] This skyline model allowed the speciation rate to vary at the top and bottom of the tree, by assuming two branching process epochs each with an i.i.d. net diversification rate. The epoch boundary was fixed at 4.2 Ga, approximately the age of LUCA. The priors of this skyline model were selected such that the root height was *a priori* centered around a 95% credible interval of 4.46 – 5.70 Ga. We used resub (with the WY, IV, EQ, DN, and SG cherries respetively) and the Null model ($\mathbb{I}_s = 0$), each with the following priors:

- Pre-LUCA net diversification rates; one per Class $\lambda - \mu \sim \text{LogNormal}(\text{mean} = 5, \sigma = 0.1)$
- Post-LUCA net diversification rates; one per Class $\lambda - \mu \sim \text{LogNormal}(\text{mean} = 10, \sigma = 1)$
- Reproduction numbers; one per Class $\frac{\lambda}{\mu} = 2$
- Sampling proportion; one per Class, fixed at 10^{-8} due to absence of sampled ancestors
- Gradual relaxed clock standard deviations; one per Class $\sim \text{Gamma}(\alpha = 5, \beta = 0.05)$
- Clock rate; amino acid substitutions per site per billion years $\sim \text{LogNormal}(\text{mean} = 0.1, \sigma = 1)$
- Relative mutation rates; one per Class $\sim \text{LogNormal}(\text{mean} = 1, \sigma = 1)$
- Gamma rate heterogeneity shapes; one per Class $\sim \text{LogNormal}(\text{mean} = 2, \sigma = 0.5)$
- Amino acid equilibrium frequencies $\sim \text{Dirichlet}(\alpha_A = 4, \alpha_C = 4, \dots, \alpha_Y = 4)$
- Amino acid exchangeability rates fixed to the aaRS empirical model
- Transition proportion $\nu \sim \text{Beta}(\alpha = 5, \beta = 5)$; parameter is not being used in the Null model
- Relative transition age $\frac{t_e - t_a}{t_h} \sim \text{Beta}(\alpha = 2, \beta = 6)$, where t_h is tree height, and t_a is the estimated height of the cherry aaRS ancestor; note that t_e is not being used in the Null model
- Model indicator $\mathbb{I}_s = \begin{cases} 0 & \text{w.p. } 0 \\ 1 & \text{w.p. } \frac{1}{2} \\ 2 & \text{w.p. } \frac{1}{4} \\ 3 & \text{w.p. } \frac{1}{4} \end{cases}$ when using resub, or fixed to 0 in the Null model

References

[1] Mendes FK, Bouckaert R, Carvalho LM, Drummond AJ (2025) How to validate a Bayesian evolutionary model. Systematic Biology (1):158–175.

[2] Douglas J, Zhang R, Bouckaert R (2021) Adaptive dating and fast proposals: Revisiting the phylogenetic relaxed clock model. PLoS computational biology 17(2):e1008322.

[3] Kass RE, Raftery AE (1995) Bayes factors. Journal of the american statistical association 90(430):773–795.

[4] Stadler T (2010) Sampling-through-time in birth–death trees. Journal of theoretical biology 267(3):396–404.

[5] Douglas J, Bouckaert R, Harris SC, Carter Jr CW, Wills PR (2025) Evolution is coupled with branching across many granularities of life. Proceedings B 292(2047):20250182.

[6] Stadler T, others, Drummond A (2013) Birth–death skyline plot reveals temporal changes of epidemic spread in HIV and hepatitis C virus (HCV). PNAS 110(1):228–233.
